# Immune Checkpoint Inhibitors: Cardiotoxicity in Pre-clinical Models and Clinical Studies

**DOI:** 10.3389/fcvm.2021.619650

**Published:** 2021-02-03

**Authors:** Shirley Xu, Umesh C. Sharma, Cheyanna Tuttle, Saraswati Pokharel

**Affiliations:** ^1^Division of Thoracic Pathology and Oncology, Department of Pathology, Roswell Park Comprehensive Cancer Center, Buffalo, NY, United States; ^2^Department of Medicine, Jacob's School of Medicine and Biomedical Sciences, University at Buffalo, Buffalo, NY, United States

**Keywords:** cardio-oncology, immunotherapy, PD-1—PDL-1 axis, immune check inhibitor (ICI), immune related adverse effects

## Abstract

Since the approval of the first immune checkpoint inhibitor (ICI) 9 years ago, ICI-therapy have revolutionized cancer treatment. Lately, antibodies blocking the interaction of programmed cell death protein (PD-1) and ligand (PD-L1) are gaining momentum as a cancer treatment, with multiple agents and cancer types being recently approved for treatment by the US Food and Drug Administration (FDA). Unfortunately, immunotherapy often leads to a wide range of immune related adverse events (IRAEs), including several severe cardiac effects and most notably myocarditis. While increased attention has been drawn to these side effects, including publication of multiple clinical observational data, the underlying mechanisms are unknown. In the event of IRAEs, the most widely utilized clinical solution is administration of high dose corticosteroids and in severe cases, discontinuation of these ICIs. This is detrimental as these therapies are often the last line of treatment options for many types of advanced cancer. In this review, we have systematically described the pathophysiology of the PD-1/PD-L1 axis (including a historical perspective) and cardiac effects in pre-clinical models, clinical trials, autoimmune mechanisms, and immunotherapy in combination with other cancer treatments. We have also reviewed the current challenges in the diagnosis of cardiac events and future directions in the field. In conclusion, this review will delve into this expanding field of cancer immunotherapy and the emerging adverse effects that should be quickly detected and prevented.

## Introduction

**Figure 1:**
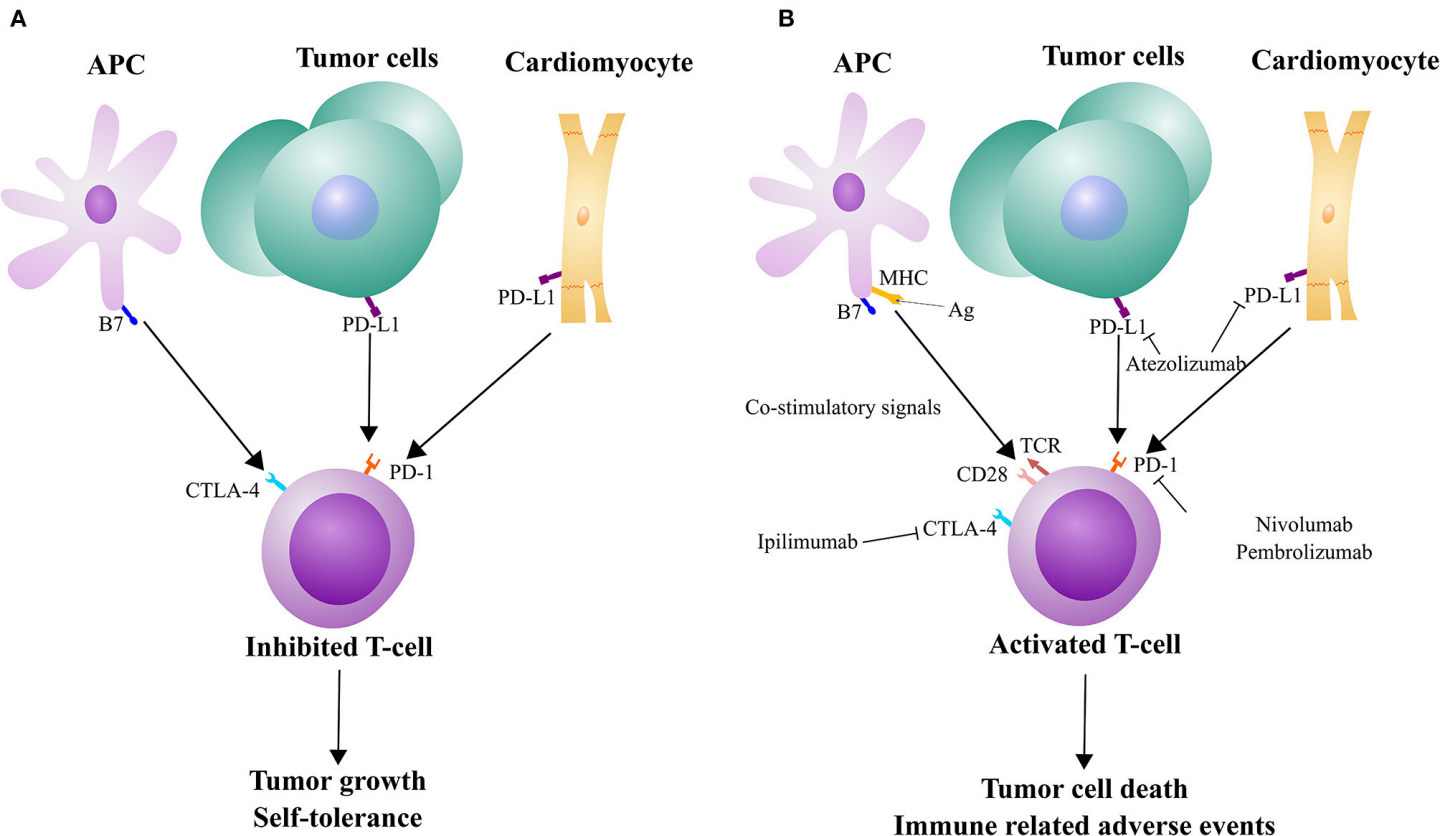
Mechanisms of tumor evasion, immunotherapy, and cardiac autoimmunity in relation to immune checkpoints. **(A)** T-cells can receive inhibitory signals from CTLA-4 and PD-1/PD-L1 that tumor cells can also take advantage of. However, the pathways are also used to maintain self-tolerance. **(B)** T-cell activation can occur with co-stimulation from MHC/TCR and B7/CD28 interactions and immune checkpoint inhibitors. While these T-cells can be used to kill tumor cells, this therapy may come at the expense of immune-related adverse events, such as against cardiac cells.

Immunotherapy is an emerging avenue for targeting cancer, particularly for difficult to treat cancers, like melanoma and non-small cell lung cancer (1). As a target for one type of ICI, some tumor cells express PD-L1 on their surface to evade detection by T-cells, which have the corresponding receptor PD-1 (Figure 1A). Monoclonal antibodies designed against PD-1 and PD-L1 are used to block that interaction, allowing for T-cell activation, ultimately leading to tumor cell death (Figure 1B). However, PD-L1 is also expressed by other, non-immune cells to maintain self-tolerance (Figure 1A) (2). There have been a wide range of different IRAEs, which are often reversible and associated with treatment response in most patients. More severe forms of IRAEs have been recorded, mostly with combined ICI medications. These IRAEs have led to the discontinuation of therapy in nearly 40% patients (2). Skin and gastrointestinal reactions are common but one of the most severe side effects is myocarditis. While the rate of early onset myocarditis in clinical trials was rare (<1% incidence at the time of FDA approval), the frequency increased with combination immunotherapy (2–4). There have also been reports of several other cardiotoxic effects, including pericarditis and myocardial infarction (4). Drobni et al. (5) reported a three-fold increased risk of atherosclerotic cardiovascular events with ICI use. While steroids are commonly used in the management of IRAEs, immunosuppressive agents are indicated for severe or steroid-refractory cases. Still, based on the severity of symptoms, IRAEs can lead to delay or discontinuation of ICI therapy (6). As these ICIs are being increasingly utilized in cancer patients, the rate of myocarditis has also increased (7).

Despite the rise of ICI-induced myocardial diseases, there is a gap in knowledge related to the pathological mechanisms. Several pre-clinical studies have attempted to model the clinical findings but produced discrepant results. Although PD-1 knockout mice display different cardiomyopathies (8, 9), cardiac inflammation has been difficult to model in animals with only PD-1/PD-L1 antibodies. In this review, we will cover the basic pathophysiology of PD-1/PD-L1 blockade, pre-clinical models, early clinical trials, recent combination therapy protocols, and proposed autoimmune mechanisms for these pathologies. Finally, we will conclude with current challenges diagnosing these cardiac events.

### Basic Pathophysiology of PD-1/PD-L1 Axis

The PD-1 receptor was discovered in a screen of genes associated with apoptosis and found to be inducible on T- and B-cells (10, 11). In a knockout mouse, the animals developed autoimmune effects that varied in organs and mouse strains (12). Nishimura et al. predicted that PD-1 suppresses the immune system for self-tolerance. The ligand PD-L1 was found by Dong et al. (13) to negatively regulate T-cells. However, the ligand was not elucidated to interact with the PD-1 receptor until later by Freeman et al. (14) Along with the other ligand PD-L2 (15), these groups found PD-L1 displayed on antigen presenting cells (APC) and tumor cells (16, 17).

Functionally, tumor cells can evade immune detection by displaying PD-L1 on the surface of their cells via “innate” and “adaptive” responses (18). Ongoing studies are also investigating PD-L1 expression as a predictor of ICI efficacy or future tumor relapse. Innate, constitutive expression differs from adaptive, downstream effects of inflammatory cytokines. While some cancer cell lines, such as melanoma, often do not constitutively produce PD-L1 *in vivo*, expression is stimulated significantly by interferon-γ (IFN-γ) and with interleukin-1α (IL-1α) and IL-27 (19). These cytokines influence expression via distinct signaling pathways that may work synergistically and allow for tumor resistance to immunotherapy treatments.

By blocking this PD-1/PD-L1 interaction through ICIs, immune cells (such as cytotoxic T-cells) are allowed to attack tumor cells. In that regard, immune cell infiltration in the tumor microenvironment may affect the efficacy of immunotherapy (20). Some tumors have little to no immune infiltration, which may be due to lack of immune cell trafficking to the site (21). PD-L1 is often upregulated in tumors in response to inflammation in an attempt to neutralize the immune cell response. As a result, the infiltrating immune cells exhibit a phenotype of decreased function, including expression of negative regulators like PD-1, where T-cells are present but have reduced cytotoxic function (21). Overall, there are many interactions between the tumor and immune cells that either enhance or hinder ICI therapy.

The PD-1/PD-L1 axis has progressed rapidly from its discovery to cancer treatment. By employing the immune system against tumor cells, this innovative therapy can be utilized for a number of cancer types. However, IRAEs and autoimmune events were observed even early on in pre-clinical experiments.

### Early Pre-clinical and Clinical Studies

Subsequent work on the PD-1 receptor found that knockout had varying effects based on the background of the mice. Interestingly, PD-1 knockout in C57BL/6 mice displayed a normal phenotype (22). However, in a BALB/c knockout, Nishimura et al. (12) observed dilated cardiomyopathy, reduced cardiac function, and antibody deposition on cardiomyocytes. Further images from Okazaki et al. (8) showed mouse serum cross-reactivity against rat tissue cardiac troponin I (cTnI). Administration of cTnI antibodies to wild-type mice also led to decreased ejection fraction (EF) and increased calcium current *in vitro*.

In the more autoimmune susceptible MRL mouse strain, PD-1 knockout led to fatal myocarditis and the formation of autoantibodies against cardiac myosin (9). The MRL mouse phenotype simulates disease in the same tissues as humans with lupus, except the mice lack overt heart disease, increasing the importance of this finding (23). This could explain why ICI therapy is poorly tolerated by patients with pre-existing autoimmune diseases (3). However, patients with these conditions are excluded from clinical trials and the benefits vs. risks should be considered with an individual's disease progression (24). With the MRL mice, Wang et al. (9) proposed that mouse fatalities were due to congestive heart failure as a result of heart dilation. They also found T-cells in the heart and PD-L1 expressed on cardiomyocytes.

In contrast, Grabie et al. (25) found that endothelial PD-L1 expression was upregulated in their induced model of CD8^+^ T-cell-mediated myocarditis. Blockade of PD-L1 led to increased cardiac inflammation and circulating cTnI. PD-L1 has been found in non-immune cells and the expression in either cardiomyocytes or endothelial cells supports self-tolerance and the progression of cardiotoxic IRAEs.

Yet due to strain variability in mice, the low rate of myocarditis, and lack of diagnostic modalities in both animals and patients, it is difficult to induce and detect these adverse cardiac effects in pre-clinical models. Additionally, humans and mice are exposed to different antigens, limiting the ability to model IRAEs. In response, an option is to lower immune self-tolerance in mice through genetic modifications (26).

In clinical trials, the occurrence of myocarditis in clinical trials was rare but may be underestimated from lack of routine diagnostic imaging and cardiac monitoring. In an early analysis of ICI-induced myocarditis, 46% of patients also experienced a major adverse cardiac event (MACE), including cardiac arrest or death. Many cases had abnormal electrocardiogram, increased levels of cardiac troponin, and reduced left ventricular EF below 50% (27). Clinical biopsies and autopsies have confirmed cases of immunotherapy-related myocarditis and observed T-cells (27) and macrophages infiltrated in the heart (3). Similarly, in a mouse model, PD-1 blockade significantly increased cytotoxic T-cells in the myocardium (28). In another mouse study, PD-1 antibodies influenced macrophage infiltration and subsequent M1 polarization (29). However, translational discrepancies between mouse models and the clinical presentation of IRAEs hinder mechanistic research and possible therapeutic options. Finally, in this review, we will investigate the adverse effects of multimodality treatment and autoimmune mechanisms.

### Autoimmune Side Effects in Combination With Radiation Therapy

Radiation and immunotherapy have been separately utilized to treat cancer, but both have limiting side effects on several organs, including the heart. As with other cancer treatments, immunotherapy with radiotherapy is being investigated as a more efficacious treatment option than monotherapy. Although the combination of radiation and immunotherapy is not currently common in practice, it may become more prevalent as ICIs gain more use as front-line treatment options. Some researchers have studied the cardiac side effects of immunotherapy in subsets of patients who underwent prior radiation exposure. While the disease response to ICI was found to be better in irradiated patients, ICI-related pulmonary toxicity was significantly higher in this cohort. The cardiotoxic potential of such combination therapy warrants further studies (30).

Animal and clinical studies show synergistic efficacy of combination therapy with radiation and ICIs (28, 30, 31). Researchers have also observed an abscopal effect where the treatment is potent against even non-irradiated tumor cells (30, 31). Other data supported that ablative radiation has anti-tumor effects dependent on cytotoxic T-cells (32). While this combination therapy may enhance anti-tumor effects (31), it comes at the risk of cumulative cardiotoxicity. Combined with radiation, PD-1 blockade appears to exacerbate radiation-initiated cardiac inflammation (28). Cumulative cardiotoxicity and inflammation have been observed in mouse models with thoracic irradiation and PD-1 blockade (33). However, radiation targeted to the lungs and PD-1 antibody did not cause mortality, suggesting cardiac inflammation as the primary mediator (28). Radiation-induced heart disease has been characterized with fibrosis and acute production of inflammatory cytokines (34) and can be compounded with ICI-induced cardiac dysfunction.

The presence of PD-L1 on non-immune cells is associated with self-tolerance from immune cells (2). Previous work has shown that PD-L1 can be induced *in vitro* on cardiomyocytes (35) and endothelial cells (36) with the addition of IFN-γ, although untreated cardiomyocytes did not have detectable levels of PD-L1 while endothelial cells had constitutive expression. In the study investigating cardiomyocytes, Seko et al. proposed IFN-γ may be produced by infiltrating immune cells in viral myocarditis. Radiotherapy itself may cause cardiac inflammation, including immune cell infiltration. In their model of viral myocarditis, PD-1 blockade increased myocardial inflammation and IFN-γ expression (35).

### Autoimmune Side Effects With Combination Immunotherapy

The FDA first approved Ipilimumab in 2011 (anti-cytotoxic T-lymphocyte associated protein-4 or CTLA-4). Since accepting Pembrolizumab and Nivolumab in 2014 (anti-PD-1) and Atezolizumab in 2016 (anti-PD-L1), these ICIs have seen expanded use in more cancer types (37–40). The agency also approved a combination immunotherapy with Ipilimumab and Nivolumab in 2015 (41). This specific combination of ICIs has been shown to be more efficacious than monotherapy in several clinical trials for melanoma, non-small cell lung cancer, and colorectal cancer (42–45). While the use of multiple immunotherapy agents has gained traction, it often comes at the expense of additional toxicity. In an analysis on Vigibase of ICI case reports from global WHO data, Wei et al. (46) reported that combination blockade against CTLA-4 and PD-1/PD-L1 had an increased rate of myocarditis than monotherapy alone (1.22% vs. 0.54%).

Combination therapy appeared to induce IFN-γ and tumor necrotic factor (TNF-α) production in mouse cardiac tissue (28). Furthermore, older treatments with CTLA-4 blockade had higher rates of IRAEs and the combination of PD-1 and CTLA-4 inhibition led to increased myocarditis cases (27). In contrast to the PD-1/PD-L1 axis, CTLA-4 is expressed on activated T-cells and leads to downstream deregulation of T-cell function. Du et al. (47) proposed that anti-CTLA-4 therapy and IRAEs have distinct mechanisms of action and showed that within the tumor microenvironment, CTLA-4 blockade locally decreases Tregs and allows other T-cells to elicit their effects. In contrast, IRAEs may be caused by activation and expansion of autoreactive T-cells in lymphoid organs. CTLA-4 knockout causes severe myocarditis, inflammation of numerous organs, and early death (48). Additionally, CTLA-4 and PD-1 utilize distinct mechanisms of the Akt pathway, which can function for synergistic tumor regulation and IRAEs (49).

Du et al. (47) were able to differentiate the mechanisms of CTLA-4 immunotherapy and adverse effects by administering Ipilimumab in a humanized *CTLA4* knock-in mouse. When this model included PD-1 antibody, severe IRAEs were observed, including inflammation of many organs. The researchers observed dilated cardiomyopathy, myocarditis, and elevated serum cTnI. The combination treatment amplified the severity of autoimmune reaction with more autoreactive T-cells and Tregs.

Recently, Wei et al. (46) created a mouse model with heterozygous CTLA-4 loss and PD-1 knockout to model clinical myocarditis, which displayed cardiac electrical abnormalities, myocardial inflammation, and increased mortality. Interestingly, they used Abatacept (a recombinant CTLA-4 and IgG fusion protein) to rescue the inflammation and prevent mortality in mice. However, the clinical utility of this approach will warrant further studies, especially considering its unknown effects on tumor growth.

Combination therapy against both PD-1 and PD-L1 can also have negative consequences. Liu et al. (50) observed fulminant cardiotoxicity from sequential PD-1 and PD-L1 blockade in a patient with lung adenocarcinoma. A similar treatment protocol in mice showed an increased anti-tumor effect but at the expense of myocardial injury and lesions. Other studies have observed increased expression of PD-L1 in injured cardiomyocytes in attempts to attenuate T-cell infiltration following inflammation (3, 51). In response, Liu et al. proposed that concurrent or subsequent blockade of PD-L1 after injury may exacerbate inflammation and lead to fulminant myocarditis.

### Mechanisms of Autoimmunity and Potential Autoantigens

Previously, patients with prior autoimmune diseases were not included in immunotherapy clinical trials. Retrospective studies have shown that there is a similar or slightly higher rate of IRAEs with these patients, although the effects are often manageable without the need to end ICI treatment (52–54). Current ongoing clinical trials (NCT03816345) will aid in elucidating the safety of immunotherapy (specifically Nivolumab) with a larger sample size and patients with a spectrum of autoimmune diseases.

In a mouse model, experimental autoimmune myocarditis (EAM) was induced by injecting a fragment of cardiac myosin to induce inflammation (55). Tsuruoka et al. observed more severe adverse effects with subsequent PD-1 blockade, including increased immune cell infiltration and expression of interleukins, collagen, and PD-L1. However, cardiac function was not significantly changed. In contrast, concurrent PD-1 antibody administration appeared to decrease CD4^+^ cell counts and there were no changes in the expression of previously observed genes. As the authors concluded, this data may support that prior autoimmune deficiencies can affect the presence of subsequent IRAEs.

At present, there are also different proposed mechanisms as to whether T-cell reactivity to antigens or autoantibody formation play a role in IRAEs (24). While the presence of immune cell infiltration, most often T-cells, is confirmation of myocarditis, previous animal and clinical data also support the role of autoantibodies. Another hypothesis is a “shared” antigen between cardiomyocytes and tumor cells that stimulates the T-cell infiltration of the myocardium (56). In addition, autoreactive T-cells (some sensitive to cardiac antigens) may be unintentionally released into the periphery from maturation in the thymus (57).

Elevated cardiac troponin levels in serum are a common biomarker for myocardial injury and cardiac troponin T (cTnT) was previously found to be elevated in a majority of ICI-induced myocarditis cases (27). Interestingly, autoantibodies against cTnT were observed in a patient with fatal Pembrolizumab-mediated myocarditis (58). Two further patient samples of ICI-mediated myocarditis displayed deposition of IgG in foci and the presence of autoimmune-related Th17 cells (59). In one of those patients, there was also an increased expression of autoimmunity-associated genes and serum presence of an immunogenic cTnI peptide and IgG against that sequence. However, not all cases of ICI-mediated myocarditis have observed similar IgG deposition. Bockstahler et al. proposed a potential mechanism where self-tolerance is abrogated by PD-1/PD-L1 blockade. Subsequent tissue damage releases self-antigens, such as cardiac troponin or myosin from cardiomyocytes. The authors hypothesized that antigen presentation by dendritic cells and inflammatory cytokines can lead to increased self-reactive CD4^+^ T-cells. These cells can then differentiate to Th17 effector cells, thereby decreasing Tregs and supporting the formation of autoantibodies.

Based on observations of IgG deposition against cTnI and cardiomyocytes in previous mouse models, Bockstahler et al. (59) injected mice with immunogenic cTnI peptide to create a model of EAM. Similar to patient samples, there were leukocyte infiltration and fibrosis in the myocardium. While autoimmune mechanisms involving self-antigens and antibodies are being investigated, modified mouse models are being developed to resemble patient presentation. In addition, new multimodality treatment protocols are utilized in clinical settings and simulated in animals with often synergistic positive and negative effects.

## Discussion

At present, ICI-related myocarditis is difficult to detect in patients, even with serial testing of cardiac troponin levels. cTnT was previously found to be elevated in a majority of ICI-induced myocarditis cases (27). However, in a study of ICI-treated non-small cell lung cancer cases, most patients with detectable cTnI concentration could be linked to a pre-existing heart disease. One patient had no previously diagnosed heart disease and was hypothesized to have benign, Nivolumab-induced myocarditis (60). In their conclusion, Sarocchi et al. supported the use of cardiac troponin testing with an emphasis on baseline conditions and pre-existing disease.

In contrast, many cases do not display decreased LVEF but a recent study observed decreased global longitudinal strain (GLS), with or without preserved EF, that correlated with MACE (61). While endomyocardial biopsies are definitive (but invasive) confirmations of myocarditis, cardiac magnetic resonance imaging (MRI) and strain analysis (calculated from MRI) could also be used to detect abnormalities (62). As ICIs are gaining more utility in a number of cancer types, there will be a growth in the patient population being treated and therefore the cases of myocarditis. It will be necessary to increase screening procedures and research into the pathophysiology of such adverse side effects. Sensitive cardiac troponin tests can detect initial, subtle changes in the heart but abnormalities should be further investigated with diagnostic imaging, as shown in Table 1 (63). Echocardiogram can detect regional wall motion abnormalities or decreased systolic function; however, these abnormalities are detected only in a subset of patients (27). Cardiac MRI is superior to echocardiography, allowing tissue characterization of myocardial edema, inflammation, and fibrosis. Because ICI-related myocarditis could lead to the discontinuation of immunotherapy, tissue diagnosis from an endomyocardial biopsy is still recommended whenever feasible (64).

**Table 1:** Diagnostic considerations for ICI-induced myocarditis and cardiotoxic events.

1. Assessment of risk factors	Prior cardiovascular events and risk factors for atherosclerotic coronary artery disease
2. Biomarker analysis	• Baseline and serial cardiac troponin I • B-type natriuretic peptide
3. Diagnostic testing and imaging	• Electrocardiogram for abnormal PR, QRS and QTc intervals, and evidence of atrial and ventricular arrhythmias • Echocardiogram to assess cardiac function and myocardial wall motion • Cardiac MRI for increased T2-signal intensity, and abnormal early and late gadolinium signal intensity in contrast-enhanced MRI • Tagged cine MRI for strain analysis
4. Endomyocardial biopsy	• Tissue edema • Lymphocyte and macrophage infiltration

Another obstacle to characterizing these adverse side effects is the lack of robust animal models. PD-1 knockout mice have shown variable phenotypes but some of the seminal work on the PD-1/PD-L1 axis observed a range of autoimmune and cardiac defects. Currently, multimodality treatment options have gained traction for cancer therapy but may also have synergistic, negative side effects. While these side effects are being investigated in clinical trials, radiation and combination immunotherapy protocols could also be utilized in animal models. Based on the clinical experience, a combination immunotherapy treatment could better exhibit ICI-induced myocarditis in an animal model. There is also the utility of genetic models, including recent advancements by Wei et al. (46) to create a CTLA-4 and PD-1 genetically altered mouse model which showed myocarditis in a similar pattern as seen in patients. Overall, the diagnosis, pathology, and treatment of IRAEs is complex and requires more research as immunotherapy matures into a more common cancer treatment option.

## Author Contributions

SX took the lead in writing the review while CT contributed sections of the Early Pre-clinical and Clinical Studies. UCS conceived the review subject and outline while both UCS and SP provided feedback and additional references. UCS also aided the creation of Table 1 and clinical considerations. UCS and SP provided feedback and additional references. All authors contributed to the article and approved the submitted version.

## Conflict of Interest

The authors declare that the research was conducted in the absence of any commercial or financial relationships that could be construed as a potential conflict of interest.
